# Reduced postural stability in men and women aged 55–65 following 14 days of head-down bed rest

**DOI:** 10.1038/s41598-025-21828-3

**Published:** 2025-10-30

**Authors:** Jeremy Rabineau, Roxanne Fournier, Eric T. Hedge, Carmelo J. Mastrandrea, Richard L. Hughson

**Affiliations:** 1 Department of Kinesiology and Health Sciences, University of Waterloo, Waterloo, ON Canada; 2 Schlegel-UW Research Institute for Aging, Waterloo, ON Canada

**Keywords:** Late middle-aged adults, Risk of falls, Balance control, Posturography, Inactivity, Microgravity, Ageing, Neurophysiology, Geriatrics, Public health

## Abstract

Aging and head-down bed rest (HDBR) decrease postural stability. Chances of being bedridden increase with age, but HDBR studies usually focus on young men. Here, we evaluate the impact of HDBR on postural stability among late middle-aged individuals. Twenty-two healthy participants (55–65 years old, 11 women) were exposed to 14-day HDBR. Eleven participants performed daily exercise. Static posturography data were collected before, 5 h after, and 4 weeks after HDBR. No time×group or time×sex effects were observed, but women had lower postural stability. With eyes open, the root mean square of the center of pressure was larger immediately after HDBR only in the medio-lateral axis (median [interquartile range]: +53% [+ 15%; +129%], *p* = 0.002). The mean velocity was increased on both axes (+ 20% [+ 8%; +46%] for medio-lateral and + 19% [+ 13%; +36%] for antero-posterior, both *p* < 0.001). The complexity features and the critical time were left unchanged. The effects of HDBR were more visible in the eyes open condition and the deconditioning was reversible after four weeks. 14-day HDBR decreased postural stability in individuals aged 55–65, with no impact of the chosen countermeasure. While the deconditioning was equivalent to two decades of aging for some features, additional research is required to determine whether age was an aggravating factor.

## Introduction

Healthy aging leads to a multi-system deconditioning that includes sensory systems degradation, musculoskeletal changes, cognitive decline, and motor control alterations^1^, ultimately resulting in decreased postural stability^2,3^. This reduced ability to maintain balance is a major concern in aging populations, as it leads to an increased risk of falls. Various large-scale studies indeed report that one fourth to one third of people over 65 years of age fall each year, with prevalence increasing with age^4,5^. Falls not only lead to major lacerations, fractures, brain injuries, or even death in older populations, but they also represent a large economic cost for the society^6,7^.

Postural stability is also altered among astronauts experiencing short-^8^ and long-term^9^ gravity unloading, as well as among participants exposed to different ground models of microgravity^10,11^. Indeed, the removal of gravity loading leads to adaptations in the multisensory integration, alteration of the neuromotor skills, and loss of force in the major postural muscles^9^. Immediately post-spaceflight, a larger contribution of the hip-strategy to maintain balance is observed, and the risk of falls is increased^8,9^. This situation is even more problematic as both (simulated) microgravity and aging induce a decrease in bone mineral density^12–14^, which increases the risk of fracture on falling^15^. Interestingly, the changes in postural stability following exposure to microgravity are reversible within a few days, but longer duration of exposure leads to longer recovery time^16^.

Six-degree head-down bed rest (HDBR) is a model used to evaluate the effects of inactivity, exposure to weightlessness, and multi-system aging^17^. While many studies report that exposure to HDBR impairs postural stability, even after only 5 days of exposure^18^, most of these studies were conducted on healthy young men^11^. So far, only one study seems to have studied the effect of HDBR on postural stability among late middle-aged adults, even though their participants were all men^19^. However, sex and age may influence postural stability^3,20^ and the severity of HDBR-induced deconditioning^21^. For instance, muscle mass and function in the lower limbs decreased more in men aged 55–65 years old compared to younger men, following the same exposure to HDBR^22,23^. Since both astronauts and bed-ridden individuals are usually older than populations typically recruited for HDBR studies, there is a real interest to better understand how age impacts microgravity- and inactivity-induced deconditioning.

Accordingly, the objective of this study, which is part of a much larger project on the multi-system impact of HDBR among late middle-aged men and women, was to evaluate the changes in static posturography features in such a population, before and after exposure to two weeks of HDBR, with or without daily countermeasure exercises (including high-intensity interval and aerobic cycling plus resistance activities). The main hypothesis was that the center of pressure (CoP) features would highlight a decreased postural stability immediately after HDBR, which would be recovered four weeks later. Since exercise countermeasures have previously shown their protective effects on postural stability both in space^24^ and in HDBR^25^, we additionally hypothesized that the postural stability in late middle-aged adults would also benefit from daily exercise countermeasures during bed rest.

## Methods

### Participants and study protocol

Twenty-two healthy late middle-aged participants (11 men, age: 60 ± 3 year, body mass: 79.8 ± 14.2 kg, body height: 173 ± 10 cm and 11 women, age: 59 ± 3 year, body mass: 60.9 ± 14.3 kg; body height: 161 ± 10 cm) were recruited to take part in this − 6° HDBR study. The inclusion criteria included being between 55 and 65 years old, as well as self-reported performance of at least 2.5 h of moderate-to-vigorous physical activity per week, lack of physical or mental illness, absence of prescribed medication, and absence of smoking in the 6 months prior to the start of the study. In addition, the female participants were required to be at least 1 year postmenopausal.

The participants stayed 26 consecutive days at the McGill University Health Centre in Montreal, Canada. Their stay included: 5 days of baseline data collection (BDC), 14 days of HDBR, 7 days of supervised recovery, and they returned 4 weeks after the end of HDBR for follow-up. During the HDBR period, the entire beds of the participants were tilted at a 6° head-down angle, and all the activities of the participants were performed in this continuous supervised 6° head-down position (with pillows permitted), including eating, sleeping, toileting, and general hygiene. The participants were allowed to freely move in bed and stretch, however, bed sensors were placed under each mattress and connected to an alarm, to ensure that the HDBR position was always maintained. They were randomly assigned to either a control (*n* = 11, including 5 women) or an exercise group (*n* = 11, including 6 women). In the control group, the participants were allowed physiotherapy exercises such as stretching, motion therapy, and massages. Meanwhile, in the exercise group, they performed three daily exercise sessions during the HDBR period, including high-intensity interval, aerobic, and resistance activities, all performed in a head-down position. The high-intensity interval and aerobic activities were performed on a cycle ergometer, while the resistance activities included lower body (hip raises, unilateral leg presses, ankle pumps, leg curls) and upper body (external rotation, lateral pull-down, chest fly, dead bug) exercises. More information regarding the study and the exact implementation of the countermeasure program is given in^26^. Such a countermeasure, described in detail in^21^, was mainly chosen to preserve cardiometabolic health^27^, maintain aerobic fitness^28^, and mitigate the negative effects of HDBR on musculoskeletal outcomes^29,30^. Importantly, the rationales to choose this countermeasure did not include its potential to preserve postural stability. Throughout this time spent by the participants at the McGill University Health Centre, the individual dietary intakes and meal macronutrient compositions were controlled based on relevant recommendations and the objective to maintain body weight^26^.

This study received approval from the McGill University Research Ethics Board (IRB00010120) and the University of Waterloo Research Ethics Board (ORE No. 40420). It was registered on ClinicalTrials.gov (NCT04964999, 16/07/2021) and was performed in agreement with the Declaration of Helsinki. Informed consent was obtained from each participant prior to starting the study. Additionally, they were made aware of their right to withdraw from the study at any time.

### Static posturography testing

Static posturography data were collected at three time points: on the second day of the baseline period (BDC), about 5 h after resuming upright posture following 14 days of HDBR (R0), and 4 weeks after the end of the HDBR period (R4wk). All sessions were performed approximately at the same time of the day, at least two hours postprandial, and with no caffeine consumption on the day of the test before the session.

The CoP dynamics in the antero-posterior (AP) and medio-lateral (ML) directions were recorded at a sampling frequency of 100 Hz, using a Wii balance board (Nintendo, Kyoto, Japan). Previous studies reported excellent test-retest reliability for this device, as well as good agreement with traditional force platform measures^31,32^.

At each time point, the participants were required to remain at least 5 min in a supine position before the test. The static posturography record itself started as soon as they moved to a standing position. The participants were requested to stand with a two-legged stance on the Wii balance board, without shoes. A researcher guided participants to position their heels 2 cm apart and their feet at a 30° angle. In addition, they were instructed to keep their arms crossed to reduce anticipatory movements^33^. The record lasted a maximum of 8 min, including 5 min with eyes open, immediately followed by 3 min with eyes closed. More information about this protocol, which also included simultaneous measures of cerebral blood flow, is provided in^34^.

### Data processing

Since the supine-to-stand transition has been shown to induce an acute reduction in cerebral blood velocity that correlates with decreased postural stability^35^, the first two minutes of the CoP signal were discarded. This means that only the last three minutes of the “eyes open condition” were kept, together with the three minutes of the “eyes closed” condition, when available. These two conditions were analyzed separately for comparison.

The post-hoc data processing was performed using Matlab (R2023a, The Mathworks, Natick, MA). Each record was first filtered using a tenth order low-pass filter with a cutoff frequency at 10 Hz. The choice of the features extracted from these CoP records was based on previous reviews targeting the discrimination between age groups and history of falls^36,37^. For each 180-second record in each condition (eyes open and eyes closed), a series of positional, dynamic, frequency, stochastic, and complexity features were computed.

#### Positional features

These features relate to the CoP positions throughout the record, without the temporal information. They thus refer to the dispersion of the trajectory, without including any dynamic aspects.

The range corresponds to the distance between the maximum and the minimum positions on a given axis. The root mean square (RMS) is the square root of the arithmetic mean of the squared distance, after centering the trajectory around its mean position. The sway area is estimated by fitting a bivariate confidence ellipse, which is expected to enclose approximately 95% of the points on the CoP path^3^.

#### Dynamic features

These features correspond to the local displacements of the CoP trajectory. They relate either to the velocity of the CoP or to the sway density, evaluating the characteristics of the CoP between one stable state and another.

The mean velocity is defined as the sum of the distances between consecutive points, also called sway length, divided by the duration of the recording. The mean velocity peak corresponds to the average absolute value of the maxima between successive zero crossing events on the CoP velocity signal^38^.

**Fig. 1:**
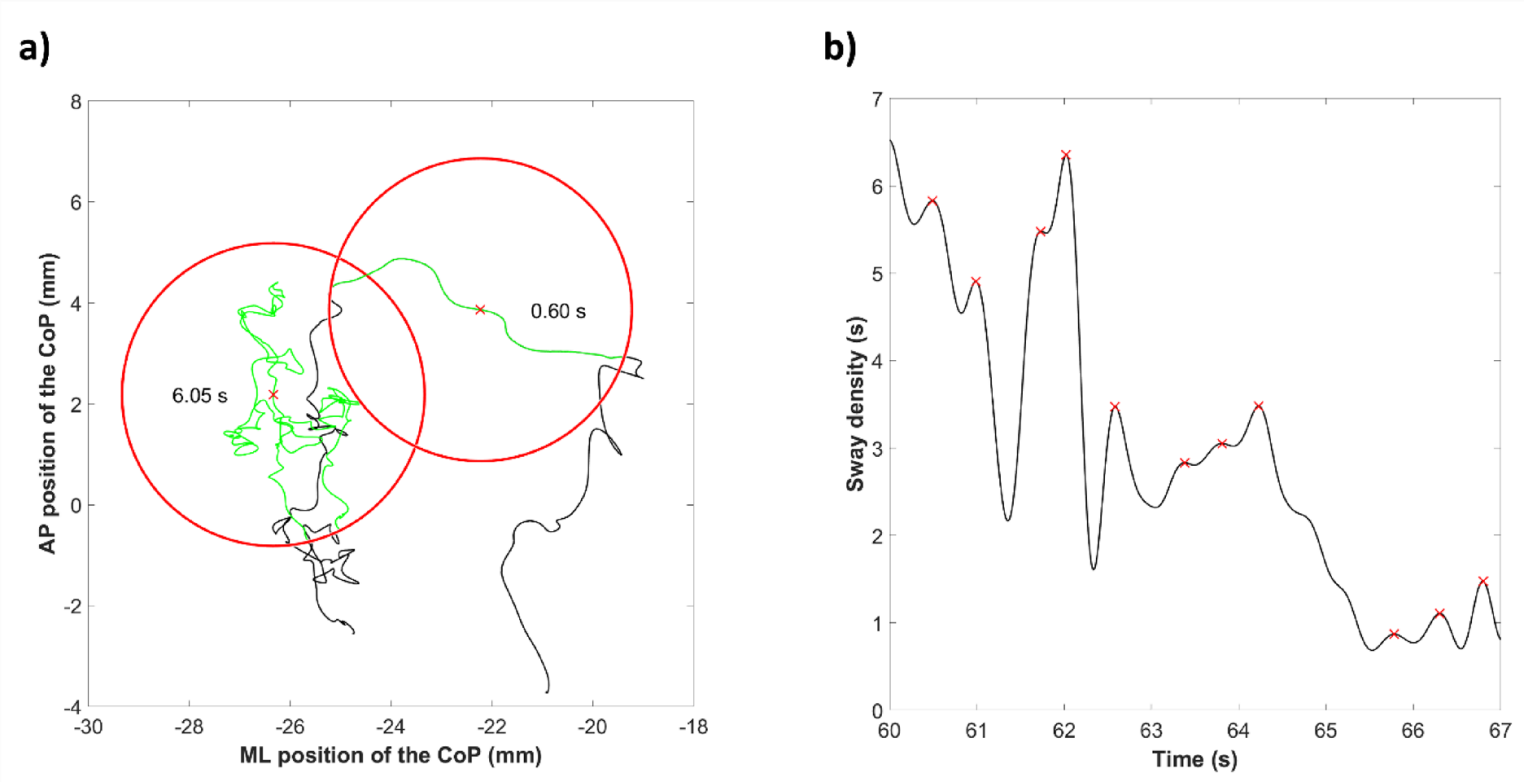
Visual representation of the principle of sway density. (**a**) Sample trajectory (black line) of the center of pressure (CoP) along the medio-lateral (ML) and antero-posterior (AP) axes. Two points (red crosses) are taken as examples for the computation of sway density: one during a period of relative stability and the other one between two periods of relative stability. The sections used for the computation of sway density are represented by green lines for each of these two points. These sections correspond to the points immediately surrounding the red crosses and falling in an area within the red circles of radius 3 mm. The numerical values of sway density are given in each red circle. (**b**) Evolution of sway density with time. The peaks of sway density are marked with red crosses and correspond to moments of relative stability. The features of interest are the mean value of a sway density peak (expressed in s), as well as the mean distance between two consecutive sway density peaks (expressed in mm).

The sway density quantifies the saccadic aspect of postural control. It consists in counting the number of consecutive samples of the CoP trajectory that fall within a circle of a given radius, and then converting this number of samples to its corresponding time interval. Here, a radius of 3 mm was chosen, as in previous studies^39^. Peaks and valleys of sway density correspond to phases of stability and destabilization, respectively^40^. Mean sway density peak was computed, as well as the mean distance between two consecutive peaks of sway density. A visual representation of the principle of sway density is given in Fig. 1.

#### Frequency features

These variables are related to the power spectral density of the CoP trajectory, estimated using Welch’s method, with 10-second segments, 50% overlapping, and linear detrending, as suggested elsewhere^41^. The total power is the energy contained in the entire power spectrum.

#### Stochastic features

These features are based on the assumption that the CoP quadratic displacement is similar to the one of a fractional Brownian motion with a short-term regime and a long-term regime^42^. The mean square displacement (MSD) was analyzed with respect to Δt in a diffusion plot, as described by Chiari and colleagues^43^.

The log-log diffusion plot was drawn to identify two linear sections defining the short- and long-term regions. Each line was defined as log(MSD) = 2 H log(Δt) + K, with K a diffusion coefficient and H a scaling coefficient. The point corresponding to the intersection between the two lines of regression is called the critical time. The latter corresponds to the transition between a short-term region of persistent movement (H > 0.5) and a long-term region of anti-persistent movement (H < 0.5).

#### Complexity features

These variables are related to the randomness of the CoP trajectory, as evaluated by the sample entropy of the Z-scored normalized velocity signal. Indeed, previous studies have shown that the difference and incremented CoP time series were better suited to this type of analysis than the regular CoP position signal^44,45^.

The velocity signal was filtered using ensemble empirical mode decomposition, with lower and upper frequencies based on previous recommendations^46^. Then, coarse-graining was performed to reach an equivalent sampling rate of 20 Hz, above which no postural information is expected to appear. The subseries length was set at m = 2, while the tolerance was set at *r* = 0.2 x WSD, where WSD was a windowed standard deviation across 240 samples. The sample entropy was computed using the code available from PhysioNet^47^.

Multiscale entropy quantifies the degree of regularity of a signal over multiple time scales^48^. Multiscale entropy was computed from sample entropy at each time scale up to 2 s, with the complexity index corresponding to the area under the curve of multiscale entropy.

### Statistical analysis

Two participants were withdrawn from the study on the third day of recovery due to health issues unrelated to the study. These participants could not complete the measurement at R4wk. In addition, the records from three additional participants were discarded due to other reasons (two had severe HDBR-induced orthostatic intolerance at R0 and one initiated voluntary hyperventilation during the protocol). Finally, some participants could not reach the end of the test at R0 due to poor orthostatic tolerance. For these participants, the data recorded with eyes closed were discarded. In the table of results, only the participants with data at all three time points were included to ensure a balanced representation. However, the statistical tests used all the data available to compare a given time point versus baseline. In the eyes open condition, 21 records were usable at BDC (10 control), 20 at R0 (10 control), and 19 at R4wk (8 control), while in the eyes closed condition, 21 records were usable at BDC (10 control), 13 at R0 (6 control), and 18 at R4wk (8 control). All the features could be computed on the usable records.

The groups and sexes were compared at baseline using Mann-Whitney tests. The combined effects of HDBR, group, and sex were evaluated using linear mixed effects models (library lme4 in R version 4.4.0) on all the data available. In the absence of interaction effects, the different time points were compared to baseline using Wilcoxon signed-rank tests, with the Bonferroni correction. Additionally, the eyes open and eyes closed conditions at a given time point were also compared using Wilcoxon signed-rank tests. Unless otherwise stated, the results are presented as median [first and third quartiles]. Statistical significance was assumed for *p* < 0.05.

## Results

The linear mixed effects models displayed no effects of group × time × sex or group × time interactions for all the features (all *p* > 0.05). Overall, women had a lower stability than men, highlighted by a larger peak velocity and mean velocity along the ML axis (both *p* < 0.05, in both eyes open and eyes closed conditions). However, no sex × time interaction effect was observed. All the results presented hereafter were thus obtained by pooling the participants of both groups and both sexes.

### Eyes open condition

#### Position features

**Fig. 2:**
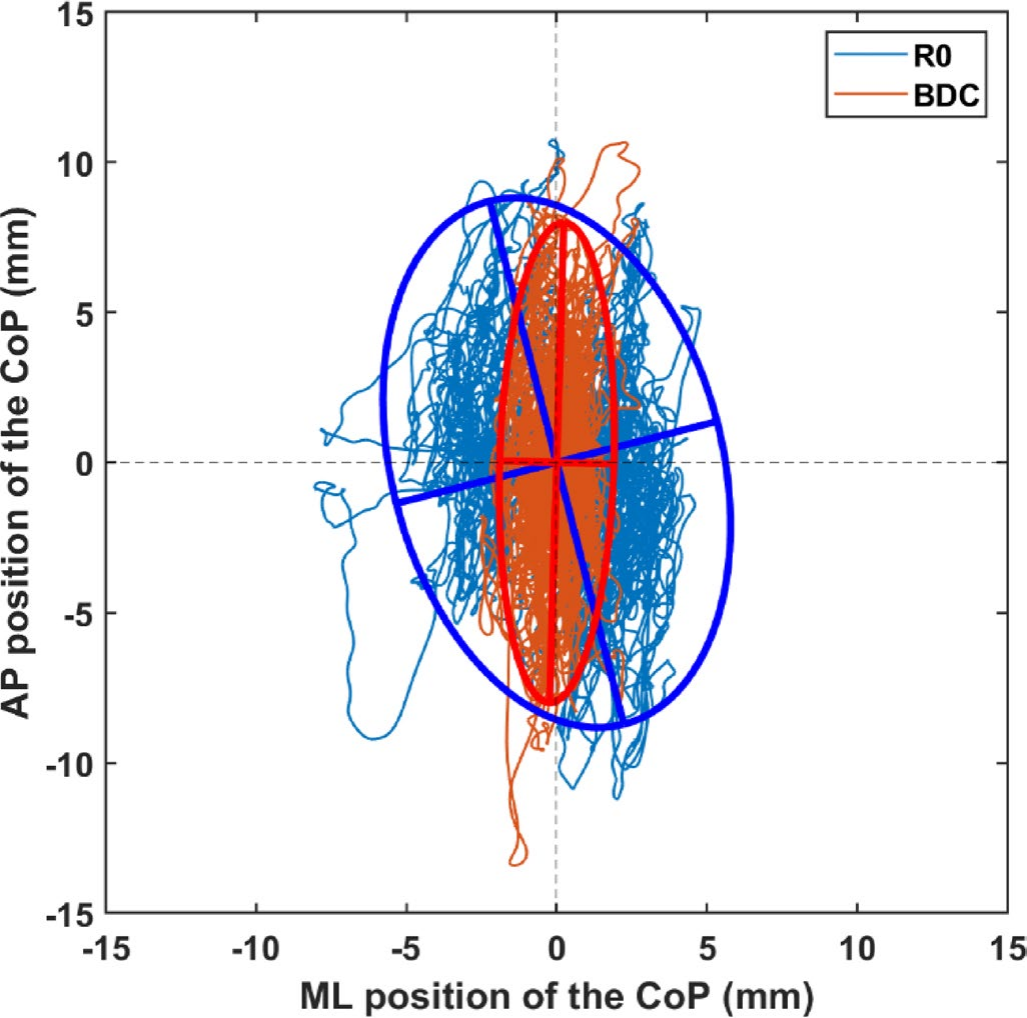
Examples of center of pressure (CoP) trajectories during the last 3 min of a 5-minute stand in eyes open condition for a representative participant. Each trajectory is centered around its mean value along the antero-posterior (AP) and medio-lateral (ML) directions. The baseline (BDC) data are displayed in red, while the data about 5 h after 14-day exposure to head-down bed rest (R0) are displayed in blue. For each time point, the 95% confidence ellipse is also plotted together with its major and minor axes.

A summary of the results for the participants who completed all three static posturography tests in the eyes open condition is presented with original units in Table 1 and here in the text as percentage change. Immediately following HDBR, both the range and RMS increased on the ML axis (+ 53% [+ 15%; +129%], *p* = 0.002 and + 22% [+ 1%; +153%], *p* = 0.012, respectively), but not on the AP axis. The area of the confidence ellipse was also increased (+ 73% [-4%; +144%], *p* = 0.023) at R0 versus BDC, caused mainly by an increase of its semi-minor axis (+ 21% [+ 0%; +115%], *p* = 0.005) rather than its semi-major axis (*p* > 0.05). These changes in the positional features can also be seen on the typical example presented in Fig. 2.

#### Dynamic and frequency features

All the dynamic features indicated a decreased stability on both axes at R0 compared to baseline, with an increase of mean velocity (+ 20% [+ 8%; +46%] along the ML axis and + 19% [+ 13%; +36%] along the AP axis, both *p* < 0.001), and mean velocity peak (+ 12% [+ 5%; +43%] along the ML axis and + 30% [+ 20%; +40%] along the AP axis, both *p* < 0.001). Meanwhile, a decrease of the mean sway density peak (-19% [-35%; -8%], *p* < 0.001) and an increase of the mean distance between sway density peaks (+ 11% [+ 3%; +24%], *p* = 0.002) were observed at R0 versus BDC. Besides this, the total power was also greater at R0 than at BDC (+ 92% [+ 2%; +187%], *p* = 0.023 on the ML axis, and + 21% [+ 0%; +68%], *p* = 0.011 on the AP axis).

#### Stochastic features

**Fig. 3:**
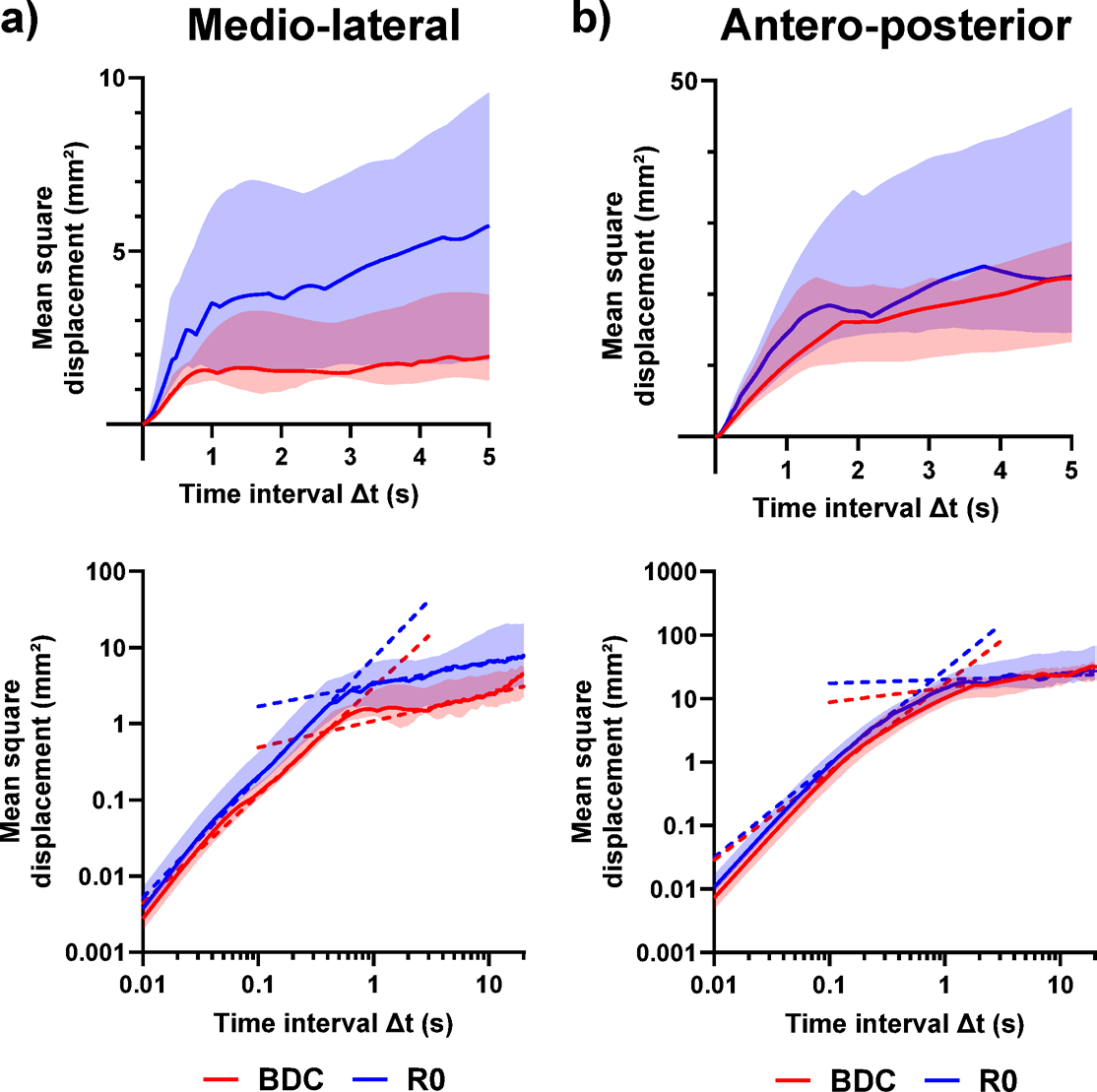
Evolution of the diffusion plots (median and interquartile range, *n* = 19) in eyes open condition before (BDC, red) and immediately after (R0, blue) 14 days of head down bed rest. The results are presented for: (**a**) the medio-lateral axis and (**b**) the antero-posterior axis. On each axis, the linear plot is represented in the top figure, while the loglog plot is represented in the bottom figure. The log-log diffusion plot identified two linear sections defining the short- and long-term regions, whose intersection happens at the critical time.

The evolution of the diffusion plots is presented in Fig. 3. It appears that the critical time was not affected by HDBR. For the AP axis, the short-term scaling coefficient was lower (less persistent) at R0 than at BDC (-0.06 [-0.09; -0.00], *p* = 0.005), which was also true for the long-term scaling coefficient (-0.05 [-0.09; -0.01], *p* = 0.023). However, no changes were observed for the scaling coefficients on the ML axis. Regarding the slopes on the log-log plots, the long-term diffusion coefficient increased on both axes (+ 0.28 [+ 0.00; +0.48], *p* = 0.016 along the ML axis and + 0.13 [+ 0.02; +0.31], *p* = 0.018 along the AP axis) and so did the short-term diffusion coefficient on the ML axis (+ 0.26 [+ 0.08; +0.43], *p* = 0.003) but not on the AP axis.

#### Complexity features

**Fig. 4:**
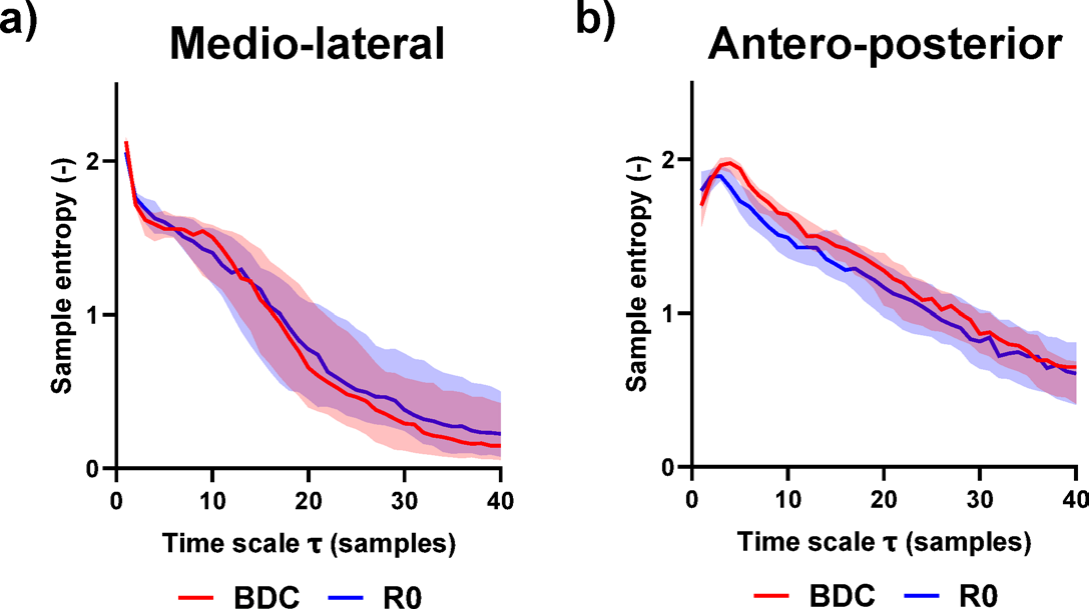
Evolution of the multiscale entropy (median and interquartile range, *n* = 19) in eyes open condition before (BDC, red) and immediately after (R0, blue) 14 days of head down bed rest. The results are presented for: (**a**) the medio-lateral axis and (**b**) the antero-posterior axis.

The sample entropy of velocity and the complexity index of velocity were preserved throughout the study. The evolution of the multiscale entropy pre- and immediately post-HDBR can be seen in Fig. 4.

All the changes observed at R0 disappeared by R4wk.

**Table 1:** Summary of the results of the static posturography tests conducted in the eyes open condition.

Type	Feature	Axis	Time point		
			BDC	R0	R4wk
**Position**	Range (mm)	AP	27.8 [22.1; 31.7]	28.4 [21.6; 39.5]	26.3 [21.1; 30.4]
		ML	10.4 [8.4; 14.9]	16.4 [12.2; 24.9] *	14.7 [8.1; 18.6]
	RMS (mm)	AP	4.5 [3.8; 5.3]	4.7 [3.5; 7.2]	4.3 [3.5; 4.8]
		ML	1.8 [1.3; 2.6]	2.7 [2.0; 3.6] *	2.0 [1.2; 2.9]
	Area of confidence ellipse (mm^2^)	/	145 [82; 241]	179 [155; 400] *	128 [73; 290]
**Dynamic**	Mean velocity (mm/s)	AP	6.2 [5.3; 7.4]	7.6 [6.7; 9.3] *	5.9 [5.1; 6.5]
		ML	4.2 [3.3; 4.5]	4.7 [4.4; 6.1] *	4.2 [3.6; 4.4]
	Mean velocity peak (mm/s)	AP	7.8 [6.6; 8.4]	9.8 [8.2; 11.8] *	6.8 [5.5; 7.7]
		ML	5.5 [4.6; 6.2]	6.2 [5.8; 7.4] *	5.2 [4.8; 6.2]
	Mean sway density peak (s)	/	3.2 [2.1; 4.1]	2.1 [1.5; 2.9] *	3.3 [2.5; 4.7]
	Mean distance between sway density peaks (mm)	/	1.9 [1.6; 2.5]	2.1 [1.8; 3.5] *	1.9 [1.6; 2.5]
**Frequency**	Total power (mm^2^)	AP	5.9 [4.1; 8.0]	6.2 [4.3; 14.7] *	6.5 [4.2; 10.4]
		ML	0.6 [0.5; 1.6]	1.4 [0.7; 5.0] *	0.9 [0.6; 1.7]
**Stochastic**	Short-term diffusion coefficient (-)	AP	1.3 [1.2; 1.6]	1.4 [1.2; 1.7]	1.3 [1.1; 1.5]
		ML	0.5 [0.4; 0.7]	0.8 [0.6; 1.2] *	0.6 [0.3; 0.8]
	Long-term diffusion coefficient (-)	AP	1.1 [1.0; 1.3]	1.2 [1.0; 1.5] *	1.1 [1.0; 1.5]
		ML	0.1 [-0.1; 0.4]	0.4 [0.1; 0.9] *	0.2 [-0.1; 0.5]
	Short-term scaling coefficient (-)	AP	0.79 [0.77; 0.82]	0.76 [0.70; 0.80] *	0.80 [0.78; 0.86]
		ML	0.72 [0.68; 0.80]	0.76 [0.69; 0.80]	0.77 [0.63; 0.82]
	Long-term scaling coefficient (-)	AP	0.12 [0.08; 0.25]	0.09 [0.03; 0.17] *	0.10 [0.01; 0.14]
		ML	0.16 [0.11; 0.18]	0.13 [0.07; 0.20]	0.16 [0.09; 0.18]
	Critical time (s)	AP	0.62 [0.43; 0.76]	0.72 [0.51; 0.89]	0.83 [0.65; 0.99]
		ML	0.55 [0.30; 0.73]	0.43 [0.32; 0.62]	0.53 [0.44; 0.64]
**Complexity**	Sample entropy of velocity (-)	AP	1.70 [1.54; 1.80]	1.77 [1.62; 1.93]	1.72 [1.57; 1.83]
		ML	2.13 [1.94; 2.15]	2.06 [1.98; 2.11]	2.11 [2.03; 2.17]
	Complexity index of velocity (-)	AP	48.6 [45.4; 51.7]	46.5 [41.7; 50.8]	50.9 [46.9; 55.9]
		ML	34.4 [24.6; 43.8]	36.5 [27.4; 43.0]	31.9 [26.9; 42.9]

*Overall*,* 21 records were usable at BDC (second day of the baseline period)*,* 20 at R0 (about 5 h after resuming upright posture following 14 days of head-down bed rest)*,* and 19 at R4wk (4 weeks after the end of the head-down bed rest period). Only the participants with data at all three time points (n = 17*,* 8 women) are included in the table to ensure a balanced representation. However*, the linear mixed effects models followed by Wilcoxon signed-rank tests used all the data available to compare a given time point versus baseline (19 for R0 vs. BDC; 17 for R4wk vs. BDC). Bonferroni correction was used to account for multiple comparisons. *: p < 0.05 compared to BDC. Exact p-values and percent changes are presented in Results.

#### Eyes closed condition

A summary of the results for the participants who completed all three static posturography tests in the eyes closed condition is presented in Table 2. Overall, most of the features suggested a lower postural stability in the eyes closed versus eyes open condition. For instance, on the AP axis during BDC testing, the mean velocity and RMS were greater (+ 78% [+ 71%; +100%], *p* < 0.001, and + 21% [+ 6%; +38%], *p* = 0.005, respectively), while the complexity index of velocity was lower (-13% [-21%; -9%], *p* < 0.001), with eyes closed versus eyes open.

Most of the changes observed in the eyes closed condition at R0 versus BDC were in the same direction as those observed in the eyes open condition. Among other results, a larger mean ML velocity and ML peak velocity (+ 26% [+ 10%; +48%] and + 29% [+ 8%; +50%], respectively, both *p* = 0.002) were seen, together with a shorter mean sway density peak (-16% [-27%; -10%], *p* = 0.017), and a larger distance between sway density peaks (+ 18% [+ 7%; +39%], *p* = 0.022). However, fewer changes were observed than in the eyes open condition, especially on the AP axis. On the latter axis, an overshoot was even observed on a few features after four weeks of recovery. For instance, a better postural stability was observed at R4wk versus BDC, as expressed by the mean AP velocity and AP velocity peak (-16% [-23%; -6%], *p* = 0.004, and − 18% [-27%; -11%], *p* = 0.003, respectively), as well as by the mean sway density peak (+ 22% [+ 2%; +58%], *p* = 0.009).

**Table 2:** Summary of the results of the static posturography tests conducted in the eyes closed condition.

Type	Feature	Axis	Time point		
			BDC	R0	R4wk
**Position**	Range (mm)	AP	37.1 [31.9; 41.2]	33.1 [27.1; 44.7]	33.6 [24.8; 57.6]
		ML	13.8 [11.9; 19.1]	16.5 [10.9; 24.0]	13.0 [9.6; 19.6]
	RMS (mm)	AP	5.3 [4.8; 6.4]	5.1 [4.0; 6.4]	5.2 [4.2; 7.7]
		ML	2.1 [1.6; 3.2]	2.6 [1.7; 3.6]	2.1 [1.4; 2.9]
	Area of confidence ellipse (mm^2^)	/	195 [153; 379]	269 [137; 417]	171 [118; 590]
**Dynamic**	Mean velocity (mm/s)	AP	13.5 [12.5; 14.9]	14.2 [11.7; 18.2]	10.6 [8.3; 13.6] *
		ML	4.6 [3.7; 5.6]	5.7 [5.0; 6.1] *	4.4 [3.9; 5.4]
	Mean velocity peak (mm/s)	AP	18.3 [16.1; 19.8]	17.9 [15.1; 25.2]	13.5 [10.3; 17.0] *
		ML	5.8 [4.9; 7.2]	7.3 [6.4; 8.0] *	5.7 [5.0; 7.0]
	Mean sway density peak (s)	/	1.2 [1.1; 1.3]	1.0 [0.8; 1.2] *	1.5 [1.0; 2.1] *
	Mean distance between sway density peaks (mm)	/	3.7 [2.8; 4.6]	4.1 [3.0; 5.9] *	2.9 [2.4; 5.5]
**Frequency**	Total power (mm^2^)	AP	14.7 [11.9; 18.8]	14.0 [11.3; 24.6]	12.0 [7.9; 33.9]
		ML	1.2 [1.0; 1.5]	1.8 [1.5; 3.5]	1.1 [0.7; 2.9]
**Stochastic**	Short-term diffusion coefficient (-)	AP	1.9 [1.8; 2.1]	1.9 [1.7; 2.2]	1.7 [1.6; 2.2]
		ML	0.8 [0.7; 1.0]	1.1 [0.9; 1.2] *	0.8 [0.8; 1.2]
	Long-term diffusion coefficient (-)	AP	1.5 [1.4; 1.6]	1.5 [1.3; 1.7]	1.5 [1.2; 1.8]
		ML	0.4 [0.2; 0.7]	0.5 [0.4; 0.8]	0.3 [0.2; 0.7]
	Short-term scaling coefficient (-)	AP	0.75 [0.70; 0.77]	0.72 [0.70; 0.80]	0.76 [0.73; 0.81]
		ML	0.74 [0.72; 0.80]	0.74 [0.71; 0.79]	0.79 [0.76; 0.83]
	Long-term scaling coefficient (-)	AP	0.12 [0.04; 0.15]	0.07 [0.04; 0.08]	0.09 [0.04; 0.16]
		ML	0.12 [0.07; 0.16]	0.09 [0.04; 0.16]	0.10 [0.04; 0.14]
	Critical time (s)	AP	0.44 [0.37; 0.60]	0.49 [0.39; 0.65]	0.58 [0.48; 0.70]
		ML	0.46 [0.38; 0.79]	0.47 [0.29; 0.55]	0.44 [0.38; 0.51]
**Complexity**	Sample entropy of velocity (-)	AP	1.56 [1.48; 1.70]	1.70 [1.56; 1.90] *	1.63 [1.39; 1.70]
		ML	2.04 [1.97; 2.11]	1.99 [1.90; 2.06]	2.04 [1.91; 2.12]
	Complexity index of velocity (-)	AP	40.8 [34.1; 45.8]	37.9 [31.1; 44.8]	42.9 [39.2; 51.0] *
		ML	35.8 [26.7; 43.7]	34.0 [26.4; 40.6]	33.7 [28.4; 41.3]

*Overall*,* 21 records were usable at BDC (second day of the baseline period)*,* 13 at R0 (about 5 h after resuming upright posture following 14 days of head-down bed rest)*,* and 18 at R4wk (4 weeks after the end of the head-down bed rest period). Only the participants with data at all three time points (n = 12*,* 3 women) are included in the table to ensure a balanced representation. However*, the linear mixed effects models followed by Wilcoxon signed-rank tests used all the data available to compare a given time point versus baseline (13 for R0 vs. BDC; 16 for R4wk vs. BDC). Bonferroni correction was used to account for multiple comparisons. *: p < 0.05 compared to BDC. Exact p-values and percent changes are presented in Results.

## Discussion

This study was the first to evaluate multiple static posturography features on late middle-aged men and women exposed to HDBR. Overall, the positional, dynamic, and frequency features all highlighted a reduced postural stability primarily in the ML axis following 14 days of HDBR, with no effect of the exercise countermeasure. Sex did not impact the HDBR-induced deconditioning, even though women had a lower postural stability throughout the study. Among the participants who could complete eyes open and eyes closed testing, the change in postural stability from BDC to R0 was more notable in the eyes open versus eyes closed condition. Unexpectedly, the critical time to switch from persistent to anti-persistent movements, as well as the complexity of the CoP signal appeared unchanged. Overall, this deconditioning was fully reversible after four weeks of recovery, with even a seemingly better stability observed on some features in the eyes closed condition.

### Effect of HDBR on postural stability

After two weeks of HDBR, the participants had poorer stability along the AP axis, highlighted by a higher mean and peak velocity, as well as a higher total power. They were, however, able to preserve their AP range and RMS at values close to baseline, at the cost of a shift toward more corrective balance control. Indeed, the lower short- and long-term scaling coefficients observed on the AP axis at R0 indicate more frequent corrective adjustments at short time scales (less persistence) and a stronger tendency to oscillate around the equilibrium rather than drifting away (more anti-persistence), respectively. On the ML axis, the same decrease in stability demonstrated by the dynamic and frequency features could not be compensated. Indeed, the persistence and anti-persistence behaviors remained similar, while the increase in both the short- and long-term diffusion coefficients highlighted a tendency for a faster drift of the CoP in both the open-loop and closed-loop phases of postural control, ultimately leading to a larger excursion along the ML axis. These results underline a clear difference between the deleterious effects of HDBR on stability along the AP and ML axes, the latter being more affected. In other HDBR studies collecting static stabilography data in younger participants, the velocity features were most commonly reported^49–51^ and the ones along the AP axis appeared to be the most sensitive to the effects of HDBR^11^, in contrast with our finding of an effect on velocity in both axes. Few studies performed stabilogram diffusion analysis in the context of HDBR. However, when this type of analysis was done, an increase of the short-term diffusion coefficient was also reported, like here^52,53^.

It is important to note that mean velocity and mean velocity peaks have both proven their sensitivity to age-related postural alterations and to history of falls^36,38,54^. More precisely, the increase in ML mean velocity observed here, after a 14-day exposure to HDBR, could be taken as equivalent to 20 [9; 45] years of typical aging^55^. Total power increased here, similarly to other HDBR studies^52^ and reports on the effect of aging on postural stability^54,56^.

After exposure to HDBR, our participants made more frequent postural corrections, as highlighted by the decrease in the mean peak of sway density, also found in younger participants after HDBR^50,51^. This is consistent with increased co-contraction indices of lower limb antagonistic muscles after HDBR^25^. Increase frequency of postural corrections^57^ and increased distance between peaks of sway density were also reported in older individuals^58^. However, these more frequent postural corrections did not limit the increased sway observed on the positional features, especially in the ML direction. Interestingly, it has been shown that the ML range was greater among fallers versus non-fallers^36^ and that RMS along the ML axis increased with age^59,60^.

The complexity features quantify the number of structural components involved in postural control as well as their coupling. These features decrease with eyes closed^44^, aging^61^, and among fallers^62^. Here, they did not change with two weeks of HDBR, suggesting that the number of mechanisms of postural control or their interaction were unchanged. Still, the gains of the different control mechanisms may have been affected by HDBR, leading to a faster drift of the CoP with time, in line with larger diffusion coefficients that may partially be explained by the decrease of lower body lean mass and muscle strength in the participants of this study^29^. Finally, the typical reaction time of these control mechanisms did not seem to be affected by HDBR, as highlighted by the absence of changes in critical time. This contrasts with the combined increased in diffusion coefficients and critical time with healthy aging^63^. Comparatively, a study recruiting adults of a similar age, found that latencies of anticipatory postural adjustments were shorter post-HDBR, while the opposite was true for the reactive response latencies^64^.

Numerous changes provoked by exposure to HDBR may have caused the observed postural stability deconditioning. A recent review describes the neuromotor changes in postural control after bed rest and lists a series of neural adaptations at the spinal and supraspinal levels^65^. These adaptations include, among others, structural brain changes, reduced spinal excitability, and increased reflex latencies. On the side of sensory receptors, exposure to HDBR or microgravity leads to ocular changes^66^ and disturbances in visual tracking^67^, as well as vestibular deconditioning^68^, and a decrease of the input of the cutaneous pressure receptors of the foot sole^69^. In addition, sensory integration is also disrupted^65^. Besides this, HDBR leads to an atrophy of anti-gravity thigh and calf muscles^70,71^, a decrease in the rate of force development^72^, an increase in the contraction time^73^, and a decrease in the stiffness of the tendon structures^74^.

### Reliance on vision

Although postural control features are reproducible with eyes open and eyes closed, the Romberg ratio has low test-retest reliability^75^. Thus, in the current study we report only features in eyes closed and eyes open conditions, and not their ratios. The comparison of Tables 1 and 2 shows that, at R0 versus BDC, a lower number of features supported a significant deconditioning of postural stability with eyes closed than with eyes open. Interestingly, in the eyes closed condition, the distinction between the ML and the AP axes was even more evident, with more changes along the former than the latter axis. Overall, these observations do not support an increased reliance on vision after exposure to (simulated) microgravity.

Postural stability is controlled by a set of redundant systems, which makes it difficult to detect a deficit in one of these systems, when participants are in an environment that is rich in sensory cues^1^. It was thus expected that posturography tests with eyes closed would be more sensitive to a decreased ability to maintain balance than their equivalent with eyes open. Decreased somatosensation and vestibular function can indeed be responsible for an increased reliance on vision to maintain a stable posture^2^. An increased reliance on visual information has already been reported with age^55^, as well as among astronauts returning from space^76^. However, in younger participants exposed to HDBR, some studies indicated a greater decline in stability with eyes closed^52^, while others reported the opposite^53,77^.

Here, importantly, a few elements may have limited the possibility to compare the eyes open and eyes closed conditions. Indeed, the whole three minutes of the eyes closed condition were analyzed, including the initial adaptation phase when switching from eyes open to eyes closed. This initial period may have induced larger sway patterns while the participant adapted to this new condition. Besides this, the whole test lasted a total of 8 min, of which the last 6 min were analyzed. Attentional drift and fatigue may have naturally caused more sway at the end (eyes closed) than at the beginning (eyes open) of this stand period. This hypothesis is supported by previous studies reporting a progressive increase of postural sway with time of stand^78^, in particular in participants with poor orthostatic tolerance^79^, which was the case here at R0^80^. A few records had indeed to be discarded at R0 due to orthostatic intolerance, which may also have introduced a survival bias in the analyses.

Additional posturography tests, such as using sway-referenced visual and/or proprioceptive cues, or standing on a soft surface, could have provided additional information regarding the integration of different sensory cues to ensure a stable posture. However, due to logistic constraints, such tests could not be performed.

### Effect of age

An overall aging effect was observed on the posturography features, which is also seen on many other physiological systems following exposure to simulated microgravity^17,81^. However, the fact that no group of younger participants was included in this study limits the possibility to assess whether age was an aggravating factor for the deleterious impact of HDBR on postural stability. In addition, the comparison of this study with other HDBR studies is not straightforward, due to the variety of features used in the literature to assess balance, including some like equilibrium scores, which may be less sensitive^11^. Unfortunately, the only HDBR study that recruited volunteers within the same age group as here, reported only positional features of static posturography^19^. Still, just like in this study, they found that 14 days of exposure to HDBR led to an increase of the RMS along the ML axis, while no or less changes were observed for RMS on the AP axis. A recent review underlines that the HDBR studies that reported changes in balance had a median length of 45 days, while those that reported no changes had a median length of 20 days^11^. Since this is the second HDBR study recruiting middle-aged older adults and observing postural changes after only 14 days, this may be a sign that postural deconditioning is faster in this population than in younger adults.

Even if some studies have shown that middle-aged older adults had a relatively similar HDBR-induced vestibular deconditioning to their younger counterparts^82^, postural control is ensured by the combination of many redundant components. As adults get older, there is an increased likelihood that some of these components of postural control function abnormally. While, among healthy participants, the central nervous system is able to compensate for small abnormalities in one or two components, the emergence of new abnormalities may affect the whole postural control process^1^. In other words, the HDBR-induced deconditioning might have a more dramatic effect on postural control for older versus younger adults. We thus encourage future HDBR studies to clearly evaluate whether age is a confounding factor for HDBR-induced balance deconditioning.

### Effect of countermeasure

Countermeasures based on physical exercise^83^, balance training^84^, and Tai Chi^85^ have already shown their efficacy at improving postural control in middle-aged and older adults. In space, most of the countermeasures currently used to tackle the deleterious effects of microgravity rely on physical exercise. They are based on specific activities that have proved useful in maintaining the postural and locomotor functions^86^. The new exercise capabilities have indeed been shown to improve the postflight agility scores, even though their main mission was to minimize the cardiovascular and musculoskeletal deconditioning^24^. Similarly, the tested countermeasure was not specifically designed to maintain postural control after HDBR^21^. While it has been shown that strength in the muscles of the lower limbs was strongly related to balance^87^, this countermeasure had no positive effect on muscle strength^29^, even though it helped preserve some features such as upper quadriceps volume^30^. Besides this, the walking speed was shown to decrease similarly in both groups after HDBR^88^.

Here, the absence of a significant effect of the tested countermeasures on postural stability agrees with some^11,50^ but not all^25^ previous studies implementing an exercise intervention during HDBR. The type of exercise chosen for this study did not target the neuromotor changes observed in postural control during HDBR. Activities such as running or jumping^25^ are indeed more challenging in terms of balance than cycling or resistive exercises. Ideally, countermeasures should act on the sensory receptors and sensory integration to have better chances to limit the HDBR-induced changes in balance^65^. For instance, foot-based vibration platforms^52^ have shown their efficacy in preserving, at least partially, postural stability. To a lesser extent it was also the case for methods based on lower-body negative pressure^49,89^ and artificial gravity^51,90^. This may be caused by the chronic stimulation of the plantar soles provided by the latter countermeasures, which has proved effective in improving postural control^61,91^.

### Reversibility

All the previous studies, conducted either with astronauts or with younger participants during HDBR, agree on a relatively quick recovery of postural stability following exposure to (simulated) microgravity^9,16,49^. Even for the most challenging tests requiring a strong engagement of the postural system, the decreased stability was no longer noticeable after 10 days^92^. Unfortunately, in this study, no intermediate data collection point was available between 0 and 28 days post-HDBR. It was thus not possible to observe the exact time course of recovery for the different postural stability features and to conclude on possible differences between older and younger participants.

Here, the overshoot observed for a few AP features in the eyes closed condition at R4wk seems to point towards a better postural stability on this axis after one month of recovery, compared to baseline. While the exact reasons behind this observation remain speculative, it may have been partially caused by the seven days of supervised recovery, immediately after the HDBR period.

### Implications

While most of the previous HDBR studies recruited younger participants^11,93^, this age group aligns more with the one of astronauts from the different space agencies^94^ and the expected age of future space tourists. Regarding terrestrial applications, the participants of this study also correspond to a population that is more likely to be bed ridden, as this probability increases with age^95^.

For astronauts on the surface of another celestial body, this decreased postural stability contributes to locomotion challenges within a bulky spacesuit and increases the risk of falls. Not only does this represent a risk to damage the spacesuit, but it also increases the risk of fracture, which is compounded by spaceflight-associated reductions in bone density^12,13^.

This decrease in bone mineral density is also observed with aging^14^, which increases the risk of fractures in case of falls^15^. Besides this, fractures often lead to a restriction of movements and cast immobilization has been shown to induce more postural instability^96^, thus increasing even more the risk of future falls^97^. More than half of adults over 60 experiencing a fall would indeed experience at least another fall within the next 12 months^98^.

### Limitations

A Wii balance board was used in this study to measure the CoP trajectory. As opposed to the laboratory force platform, which is the gold standard for such measures, this device was not strictly designed for such applications. However, the studies assessing the performance of the Wii balance board for posturography applications have reported both excellent test-retest reliability and a good agreement with traditional force platforms^31,32^.

Due to the lack of consistency in the literature regarding the methodology and features to estimate balance, it was difficult to compare the HDBR-induced postural stability deconditioning in these participants to the one of younger participants. Future studies should include groups of different ages to evaluate the interaction effect between age and HDBR.

Another limitation was the relatively low sample size, which was restricted by logistic reasons. This limited the statistical power and prevented some in-depth analyses regarding sex-based differences. The number of participants was even lower in the eyes closed condition, because of difficulties completing this test immediately after bed rest.

Body mass is a confounding factor that can have a higher influence on postural stability than age^55^. Here, the mean body mass decreased by about 1 kg due to HDBR^29^. It is highly unlikely that such small changes had a large impact on the measured features. Visual acuity is also a parameter that can influence balance^99^ and that can be affected by exposure to (simulated) microgravity^66^. However, visual acuity was not measured in this study.

In addition, a given deconditioning in the various static posturography features cannot directly be converted to a specific increase in the risk of falls^36^. Indeed, most of the falls do not occur during static standing, but rather during ambulation^100^.

Finally, care should be taken before directly extending these results to microgravity. Indeed, HDBR participants still experience the effect of a gravity vector while in bed, which differs from the situation of free-floating astronauts.

## Conclusion

Two weeks of exposure to HDBR among a group of men and women aged 55–65 years old led to a decreased postural stability. In some features, this deconditioning was equivalent to about two decades of healthy aging and may increase the risk of falls. The exact changes in the postural stability features highlighted a larger deconditioning on the ML axis than on the AP axis, which was also true for the reliance on vision to maintain balance.

Even though the amplitude of this HDBR-deconditioning was not sex-dependent, women systematically had a lower stability. It cannot be concluded whether age was an aggravating factor, even though there are reasons to believe that it should be. The chosen countermeasure, based on physical exercises, did not help preserve postural stability.

While all the changes were reversible after one month of recovery, it is crucial to find better countermeasures adapted to this age group. Such countermeasures could be applied both to astronauts and to bed-ridden patients to keep their risk of falls within an acceptable range.

## Data Availability

The data that support the findings of this study are available from the corresponding author upon reasonable request.

## References

[1] Horak, F. B., Shupert, C. L. & Mirka, A. Components of postural dyscontrol in the elderly: a review. *Neurobiol. Aging*. **10**, 727–738. 10.1016/0197-4580(89)90010-9 (1989). 2697808 10.1016/0197-4580(89)90010-9

[2] Choy, N. L., Brauer, S. & Nitz, J. Changes in postural stability in women aged 20 to 80 years. *J. Gerontol. Biol. Sci. Med. Sci.* **58**, 525–530. 10.1093/gerona/58.6.m525 (2003). 10.1093/gerona/58.6.m525 12807923

[3] Prieto, T. E., Myklebust, J. B., Hoffmann, R. G., Lovett, E. G. & Myklebust, B. M. Measures of postural steadiness: differences between healthy young and elderly adults. *IEEE Trans. Biomed. Eng.* **43**, 956–966. 10.1109/10.532130 (1996). 9214811 10.1109/10.532130

[4] Gale, C. R., Cooper, C. & Aihie Sayer, A. Prevalence and risk factors for falls in older men and women: the english longitudinal study of ageing. *Age Ageing*. **45**, 789–794. 10.1093/ageing/afw129 (2016). 27496938 10.1093/ageing/afw129 PMC5105823

[5] Bergen, G., Stevens, M. R. & Burns, E. R. Falls and fall injuries among adults aged >/=65 Years - United States, 2014. *MMWR Morb Mortal. Wkly. Rep.* **65**, 993–998. 10.15585/mmwr.mm6537a2 (2016). 27656914 10.15585/mmwr.mm6537a2

[6] Jia, H. et al. Prevalence, risk factors, and burden of disease for falls and balance or walking problems among older adults in the U.S. *Prev. Med.* **126**, 105737. 10.1016/j.ypmed.2019.05.025 (2019). 31150739 10.1016/j.ypmed.2019.05.025

[7] Crane, M. A., Lam, A., Christmas, C., Gemmill, A. & Romley, J. A. Epidemiology of mortality attributed to falls in older adults in the US, 1999–2020. *J. Am. Geriatr. Soc.* **72**, 303–307. 10.1111/jgs.18600 (2024). 37767943 10.1111/jgs.18600

[8] Speers, R. A., Paloski, W. H. & Kuo, A. D. Multivariate changes in coordination of postural control following spaceflight. *J. Biomech.* **31**, 883–889. 10.1016/s0021-9290(98)00065-7 (1998). 9840753 10.1016/s0021-9290(98)00065-7

[9] Wood, S. J., Paloski, W. H. & Clark, J. B. Assessing sensorimotor function following ISS with computerized dynamic posturography. *Aerosp. Med. Hum. Perform.* **86**, A45–A53. 10.3357/AMHP.EC07.2015 (2015). 26630195 10.3357/AMHP.EC07.2015

[10] Robin, A. et al. Comprehensive assessment of physiological responses in women during the ESA dry immersion VIVALDI microgravity simulation. *Nat. Commun.* **14**, 6311. 10.1038/s41467-023-41990-4 (2023). 37813884 10.1038/s41467-023-41990-4 PMC10562467

[11] Saumur, T. M., Gregor, S., Mochizuki, G., Mansfield, A. & Mathur, S. The effect of bed rest on balance control in healthy adults: A systematic scoping review. *J. Musculoskelet. Neuronal Interact.* **20**, 101–113 (2020). 32131374 PMC7104588

[12] Orwoll, E. S. et al. Skeletal health in long-duration astronauts: nature, assessment, and management recommendations from the NASA bone summit. *J. Bone Min. Res.* **28**, 1243–1255. 10.1002/jbmr.1948 (2013). 10.1002/jbmr.1948 23553962

[13] Spector, E. R., Smith, S. M. & Sibonga, J. D. Skeletal effects of long-duration head-down bed rest. *Aviat. Space Environ. Med.* **80**, A23–28. 10.3357/asem.br02.2009 (2009). 19476166 10.3357/asem.br02.2009

[14] Warming, L., Hassager, C. & Christiansen, C. Changes in bone mineral density with age in men and women: a longitudinal study. *Osteoporos. Int.* **13**, 105–112. 10.1007/s001980200001 (2002). 11905520 10.1007/s001980200001

[15] Mackey, D. C. et al. High-trauma fractures and low bone mineral density in older women and men. *JAMA* **298**, 2381–2388. 10.1001/jama.298.20.2381 (2007). 18042915 10.1001/jama.298.20.2381

[16] Miller, C. A. et al. Functional task and balance performance in bed rest subjects and astronauts. *Aerosp. Med. Hum. Perform.* **89**, 805–815. 10.3357/AMHP.5039.2018 (2018). 30126513 10.3357/AMHP.5039.2018

[17] Kehler, D. S., Theou, O. & Rockwood, K. Bed rest and accelerated aging in relation to the musculoskeletal and cardiovascular systems and frailty biomarkers: A review. *Exp. Gerontol.* **124**, 110643. 10.1016/j.exger.2019.110643 (2019). 31255732 10.1016/j.exger.2019.110643

[18] Mulder, E. et al. Effects of five days of bed rest with and without exercise countermeasure on postural stability and gait. *J. Musculoskelet. Neuronal Interact.* **14**, 359–366 (2014). 25198232

[19] Sarabon, N. & Rosker, J. Effect of 14 days of bed rest in older adults on parameters of the body sway and on the local ankle function. *J. Electromyogr. Kinesiol.* **23**, 1505–1511. 10.1016/j.jelekin.2013.09.002 (2013). 24099756 10.1016/j.jelekin.2013.09.002

[20] Wolfson, L., Whipple, R., Derby, C. A., Amerman, P. & Nashner, L. Gender differences in the balance of healthy elderly as demonstrated by dynamic posturography. *J. Gerontol.* **49**, M160–167. 10.1093/geronj/49.4.m160 (1994). 8014390 10.1093/geronj/49.4.m160

[21] Hedge, E. T. et al. Implementation of exercise countermeasures during spaceflight and microgravity analogue studies: developing countermeasure protocols for bedrest in older adults (BROA). *Front. Physiol.* **13**, 928313. 10.3389/fphys.2022.928313 (2022). 36017336 10.3389/fphys.2022.928313 PMC9395735

[22] Pisot, R. et al. Greater loss in muscle mass and function but smaller metabolic alterations in older compared with younger men following 2 Wk of bed rest and recovery. *J. Appl. Physiol. (1985)*. **120**, 922–929. 10.1152/japplphysiol.00858.2015 (2016). 26823343 10.1152/japplphysiol.00858.2015

[23] Tanner, R. E. et al. Age-related differences in lean mass, protein synthesis and skeletal muscle markers of proteolysis after bed rest and exercise rehabilitation. *J. Physiol.* **593**, 4259–4273. 10.1113/JP270699 (2015). 26173027 10.1113/JP270699 PMC4594296

[24] Wood, S. J., Loehr, J. A. & Guilliams, M. E. Sensorimotor reconditioning during and after spaceflight. *NeuroRehabilitation* **29**, 185–195. 10.3233/NRE-2011-0694 (2011). 22027081 10.3233/NRE-2011-0694

[25] Ritzmann, R. et al. High intensity jump exercise preserves posture Control, Gait, and functional mobility during 60 days of Bed-Rest: an RCT including 90 days of Follow-Up. *Front. Physiol.* **9**, 1713. 10.3389/fphys.2018.01713 (2018). 30559676 10.3389/fphys.2018.01713 PMC6287051

[26] Hajj-Boutros, G. et al. Myths and methodologies: Understanding the health impact of head down bedrest for the benefit of older adults and astronauts. study protocol of the Canadian bedrest study. *Exp. Physiol.*10.1113/EP091473 (2024). 38372420 10.1113/EP091473 PMC11061626

[27] Mastrandrea, C. J. et al. Exercise attenuates bed rest-induced increases in insulin resistance while alpha-klotho increases in 55 to 65 year-old women and men. *Sci. Rep.* **15**, 26927. 10.1038/s41598-025-12770-5 (2025). 40707556 10.1038/s41598-025-12770-5 PMC12290009

[28] Hedge, E. T., Mastrandrea, C. J. & Hughson, R. L. Loss of cardiorespiratory fitness and its recovery following two weeks of head-down bed rest and the protective effects of exercise in 55- to 65-yr-old adults. *J. Appl. Physiol. (1985)*. **134**, 1022–1031. 10.1152/japplphysiol.00726.2022 (2023). 36927144 10.1152/japplphysiol.00726.2022

[29] Hajj-Boutros, G. et al. Impact of 14 days of bed rest in older adults and an exercise countermeasure on body composition, muscle Strength, and cardiovascular function: Canadian space agency standard measures. *Gerontology* **69**, 1284–1294. 10.1159/000534063 (2023). 37717560 10.1159/000534063 PMC10634275

[30] Dulac, M. et al. A multimodal exercise countermeasure prevents the negative impact of head-down Tilt bed rest on muscle volume and mitochondrial health in older adults. *The J. Physiology* **603** (13), 3813–3836.10.1113/JP285897(2024). 10.1113/JP285897 PMC12306415 38878232

[31] Clark, R. A. et al. Validity and reliability of the Nintendo Wii balance board for assessment of standing balance. *Gait Posture*. **31**, 307–310. 10.1016/j.gaitpost.2009.11.012 (2010). 20005112 10.1016/j.gaitpost.2009.11.012

[32] Scaglioni-Solano, P. & Aragon-Vargas, L. F. Validity and reliability of the Nintendo Wii balance board to assess standing balance and sensory integration in highly functional older adults. *Int. J. Rehabil Res.* **37**, 138–143. 10.1097/MRR.0000000000000046 (2014). 24445863 10.1097/MRR.0000000000000046

[33] Patla, A. E., Ishac, M. G. & Winter, D. A. Anticipatory control of center of mass and joint stability during voluntary arm movement from a standing posture: interplay between active and passive control. *Exp. Brain Res.* **143**, 318–327. 10.1007/s00221-001-0968-6 (2002). 11889509 10.1007/s00221-001-0968-6

[34] Hedge, E. T., Mastrandrea, C. J., Patterson, C. A. & Hughson, R. L. Sex differences between post-menopausal women and similar age men in response to orthostatic stress following two weeks of bed rest. *J. Appl. Physiol.* **138**, 226–237. 10.1152/japplphysiol.00477.2024 (2025). 39656788 10.1152/japplphysiol.00477.2024

[35] Fitzgibbon-Collins, L. K., Noguchi, M., Heckman, G. A., Hughson, R. L. & Robertson, A. D. Acute reduction in cerebral blood velocity on supine-to-stand transition increases postural instability in young adults. *Am. J. Physiol. Heart Circ. Physiol.* **317**, H1342–H1353. 10.1152/ajpheart.00360.2019 (2019). 31674810 10.1152/ajpheart.00360.2019

[36] Quijoux, F. et al. Center of pressure displacement characteristics differentiate fall risk in older people: A systematic review with meta-analysis. *Ageing Res. Rev.* **62**, 101117. 10.1016/j.arr.2020.101117 (2020). 32565327 10.1016/j.arr.2020.101117

[37] Quijoux, F. et al. A review of center of pressure (COP) variables to quantify standing balance in elderly people: algorithms and open-access code. *Physiol. Rep.* **9**, e15067. 10.14814/phy2.15067 (2021). 34826208 10.14814/phy2.15067 PMC8623280

[38] Hewson, D. J., Singh, N. K., Snoussi, H. & Duchene, J. in *Annual International Conference of the IEEE Engineering in Medicine and Biology.* 3678–3681 (IEEE, 2010). 10.1109/IEMBS.2010.5627649 21097047

[39] Jacono, M., Casadio, M., Morasso, P. G. & Sanguineti, V. The sway-density curve and the underlying postural stabilization process. *Mot. Control*. **8**, 292–311. 10.1123/mcj.8.3.292 (2004). 10.1123/mcj.8.3.292 15322309

[40] Baratto, L., Morasso, P. G., Re, C. & Spada, G. A new look at posturographic analysis in the clinical context: sway-density versus other parameterization techniques. *Mot. Control*. **6**, 246–270. 10.1123/mcj.6.3.246 (2002). 10.1123/mcj.6.3.246 12122219

[41] Vieira, T. M., Oliveira, L. F. & Nadal, J. Estimation procedures affect the center of pressure frequency analysis. *Braz J. Med. Biol. Res.* **42**, 665–673. 10.1590/s0100-879x2009000700012 (2009). 19578647 10.1590/s0100-879x2009000700012

[42] Collins, J. J. & De Luca, C. J. Open-loop and closed-loop control of posture: a random-walk analysis of center-of-pressure trajectories. *Exp. Brain Res.* **95**, 308–318. 10.1007/BF00229788 (1993). 8224055 10.1007/BF00229788

[43] Chiari, L., Cappello, A. & Lenzi, D. Della Croce, U. An improved technique for the extraction of stochastic parameters from stabilograms. *Gait Posture*. **12**, 225–234. 10.1016/s0966-6362(00)00086-2 (2000). 11154933 10.1016/s0966-6362(00)00086-2

[44] Ramdani, S., Seigle, B., Lagarde, J., Bouchara, F. & Bernard, P. L. On the use of sample entropy to analyze human postural sway data. *Med. Eng. Phys.* **31**, 1023–1031. 10.1016/j.medengphy.2009.06.004 (2009). 19608447 10.1016/j.medengphy.2009.06.004

[45] Rhea, C. K. et al. Noise and complexity in human postural control: interpreting the different estimations of entropy. *PLoS One*. **6**, e17696. 10.1371/journal.pone.0017696 (2011). 21437281 10.1371/journal.pone.0017696 PMC3060087

[46] Gow, B. J., Peng, C. K., Wayne, P. M. & Ahn, A. C. Multiscale entropy analysis of center-of-pressure dynamics in human postural control: methodological considerations. *Entropy* **17**, 7926–7947 (2015).

[47] Lake, D. E., Richman, J. S., Griffin, M. P. & Moorman, J. R. Sample entropy analysis of neonatal heart rate variability. *Am. J. Physiol. Regul. Integr. Comp. Physiol.* **283**, R789–797. 10.1152/ajpregu.00069.2002 (2002). 12185014 10.1152/ajpregu.00069.2002

[48] Costa, M., Goldberger, A. L. & Peng, C. K. Multiscale entropy analysis of complex physiologic time series. *Phys. Rev. Lett.* **89**, 068102. 10.1103/PhysRevLett.89.068102 (2002). 12190613 10.1103/PhysRevLett.89.068102

[49] Viguier, M., Dupui, P. & Montoya, R. Posture analysis on young women before and after 60 days of -6 degrees head down bed rest (Wise 2005). *Gait Posture*. **29**, 188–193. 10.1016/j.gaitpost.2008.08.001 (2009). 18815039 10.1016/j.gaitpost.2008.08.001

[50] Kouzaki, M. et al. Effects of 20-day bed rest with and without strength training on postural sway during quiet standing. *Acta Physiol.* **189**, 279–292 (2007). 10.1111/j.1748-1716.2006.01642.x 17305708

[51] De Martino, E. et al. Intermittent short-arm centrifugation is a partially effective countermeasure against upright balance deterioration following 60-day head-down Tilt bed rest. *J. Appl. Physiol. (1985)*. **131**, 689–701. 10.1152/japplphysiol.00180.2021 (2021). 34197228 10.1152/japplphysiol.00180.2021

[52] Muir, J., Judex, S., Qin, Y. X. & Rubin, C. Postural instability caused by extended bed rest is alleviated by brief daily exposure to low magnitude mechanical signals. *Gait Posture*. **33**, 429–435 (2011). 21273076 10.1016/j.gaitpost.2010.12.019 PMC3050431

[53] Miyoshi, T. et al. Effect of long-term bedrest on lower leg muscle activation patterns during quiet standing. *J. Gravitational Physiology: J. Int. Soc. Gravitational Physiol.* **8**, P85–86 (2001). 12650185

[54] Kim, J. W. et al. Sex differences in the postural sway characteristics of young and elderly subjects during quiet natural standing. *Geriatr. Gerontol. Int.* **10**, 191–198. 10.1111/j.1447-0594.2009.00582.x (2010). 20100287 10.1111/j.1447-0594.2009.00582.x

[55] Du Pasquier, R. A. et al. The effect of aging on postural stability: a cross sectional and longitudinal study. *Neurophysiol. Clin.* **33**, 213–218. 10.1016/j.neucli.2003.09.001 (2003). 14672821 10.1016/j.neucli.2003.09.001

[56] Loughlin, P. J. & Redfern, M. S. Spectral characteristics of visually induced postural sway in healthy elderly and healthy young subjects. *IEEE Trans. Neural Syst. Rehabil Eng.* **9**, 24–30. 10.1109/7333.918273 (2001). 11482360 10.1109/7333.918273

[57] Baltich, J., von Tscharner, V. & Nigg, B. M. Degradation of postural control with aging. *Proc. Inst. Mech. Eng. H*. **229**, 638–644. 10.1177/0954411915596013 (2015). 26174561 10.1177/0954411915596013

[58] Kim, J. et al. Effects of vision, age and gender on structural and global posturographic features during quiet standing. *Int. J. Precis. Eng. Manuf.* **13**, 969–975 (2012).

[59] Piirtola, M. & Era, P. Force platform measurements as predictors of falls among older people–a review. *Gerontology* **52**, 1–16 (2006). 16439819 10.1159/000089820

[60] Swanenburg, J., de Bruin, E. D., Uebelhart, D. & Mulder, T. Falls prediction in elderly people: a 1-year prospective study. *Gait Posture*. **31**, 317–321. 10.1016/j.gaitpost.2009.11.013 (2010). 20047833 10.1016/j.gaitpost.2009.11.013

[61] Wei, Q. et al. Multivariate multiscale entropy applied to center of pressure signals analysis: an effect of vibration stimulation of shoes. *Entropy* **14**, 2157–2172 (2012).

[62] Costa, M. et al. Noise and poise: enhancement of postural complexity in the elderly with a stochastic-resonance-based therapy. *Europhys. Lett.* **77**, 68008. 10.1209/0295-5075/77/68008 (2007). 17710211 10.1209/0295-5075/77/68008 PMC1949396

[63] Collins, J. J., De Luca, C. J., Burrows, A. & Lipsitz, L. A. Age-related changes in open-loop and closed-loop postural control mechanisms. *Exp. Brain Res.* **104**, 480–492. 10.1007/BF00231982 (1995). 7589299 10.1007/BF00231982

[64] Sarabon, N. & Rosker, J. Effects of Fourteen-Day bed rest on trunk stabilizing functions in aging adults. *Biomed. Res. Int.* **2015** (309386). 10.1155/2015/309386 (2015). 10.1155/2015/309386 PMC4637013 26601104

[65] Ritzmann, R., Centner, C., Hughes, L., Waldvogel, J. & Marusic, U. Neuromotor changes in postural control following bed rest. *J. Physiol.*10.1113/JP285668 (2025). 40237347 10.1113/JP285668 PMC12810218

[66] Taibbi, G. et al. Ocular outcomes comparison between 14- and 70-Day Head-Down-Tilt bed rest. *Invest. Ophthalmol. Vis. Sci.* **57**, 495–501. 10.1167/iovs.15-18530 (2016). 26868753 10.1167/iovs.15-18530 PMC4758300

[67] Tomilovskaya, E. S., Berger, M., Gerstenbrand, F. & Kozlovskaya, I. B. Effects of long-duration space flights on characteristics of the vertical gaze fixation reaction. *J. Vestib. Res.* **23**, 3–12. 10.3233/VES-130470 (2013). 23549050 10.3233/VES-130470

[68] Yuan, P. et al. Vestibular brain changes within 70 days of head down bed rest. *Hum. Brain. Mapp.* **39**, 2753–2763 (2018). 29528169 10.1002/hbm.24037 PMC6033666

[69] Kozlovskaya, I. et al. Role of support afferentation in control of the tonic muscle activity. *Acta Astronaut.* **60**, 285–294 (2007).

[70] Belavy, D. L. et al. Differential atrophy of the lower-limb musculature during prolonged bed-rest. *Eur. J. Appl. Physiol.* **107**, 489–499. 10.1007/s00421-009-1136-0 (2009). 19680682 10.1007/s00421-009-1136-0

[71] Marusic, U., Narici, M., Simunic, B., Pisot, R. & Ritzmann, R. Nonuniform loss of muscle strength and atrophy during bed rest: a systematic review. *J. Appl. Physiol. (1985)*. **131**, 194–206. 10.1152/japplphysiol.00363.2020 (2021). 33703945 10.1152/japplphysiol.00363.2020 PMC8325614

[72] Alkner, B. A., Norrbrand, L. & Tesch, P. A. Neuromuscular adaptations following 90 days bed rest with or without resistance exercise. *Aerosp. Med. Hum. Perform.* **87**, 610–617. 10.3357/AMHP.4383.2016 (2016). 27503040 10.3357/AMHP.4383.2016

[73] Pisot, R. et al. Whole muscle contractile parameters and thickness loss during 35-day bed rest. *Eur. J. Appl. Physiol.* **104**, 409–414. 10.1007/s00421-008-0698-6 (2008). 18297302 10.1007/s00421-008-0698-6

[74] Kubo, K. et al. Effects of 20 days of bed rest on the viscoelastic properties of tendon structures in lower limb muscles. *Br. J. Sports Med.* **38**, 324–330. 10.1136/bjsm.2003.005595 (2004). 15155437 10.1136/bjsm.2003.005595 PMC1724819

[75] Tjernström, F., Björklund, M. & Malmström, E. M. Romberg ratio in quiet stance posturography—Test to retest reliability. *Gait Posture*. **42**, 27–31 (2015). 25891528 10.1016/j.gaitpost.2014.12.007

[76] Black, F. O., Paloski, W. H., Doxey-Gasway, D. D. & Reschke, M. F. Vestibular plasticity following orbital spaceflight: recovery from postflight postural instability. *Acta Otolaryngol. Suppl.* **520 Pt 2**, 450–454. 10.3109/00016489509125296 (1995). 8749187 10.3109/00016489509125296

[77] Morishima, K. et al. Effects of 20 days horizontal bed rest on maintaining upright standing posture in young persons. *J. Gravit. Physiol.* **4**, S41–45 (1997). 11541175

[78] Singh, D., Park, W., Levy, M. S. & Jung, E. S. The effects of obesity and standing time on postural sway during prolonged quiet standing. *Ergonomics* **52**, 977–986. 10.1080/00140130902777636 (2009). 19629812 10.1080/00140130902777636

[79] Claydon, V. E. & Hainsworth, R. Increased postural sway in control subjects with poor orthostatic tolerance. *J. Am. Coll. Cardiol.* **46**, 1309–1313. 10.1016/j.jacc.2005.07.011 (2005). 16198849 10.1016/j.jacc.2005.07.011

[80] Mastrandrea, C. J. et al. High-intensity exercise does not protect against orthostatic intolerance following bedrest in 55- to 65-yr-old men and women. *Am. J. Physiol. Regul. Integr. Comp. Physiol.* **325**, R107–R119. 10.1152/ajpregu.00315.2022 (2023). 37184226 10.1152/ajpregu.00315.2022

[81] Rabineau, J. et al. Cardiovascular deconditioning and impact of artificial gravity during 60-day head-down bed rest-Insights from 4D flow cardiac MRI. *Front. Physiol.* **13**, 944587. 10.3389/fphys.2022.944587 (2022). 36277205 10.3389/fphys.2022.944587 PMC9586290

[82] Gavrilin, V. & Zakharova, L. Vestibular function of middle-aged persons exposed to 30-day head-down Tilt hypokinesia. *Kosmicheskaia Biologiia I Aviakosm. Meditsina*. **19**, 15–20 (1985). 3878915

[83] Patti, A. et al. Pain perception and stabilometric parameters in people with chronic low back pain after a pilates exercise program: A randomized controlled trial. *Med. (Baltim).* **95**, e2414. 10.1097/MD.0000000000002414 (2016). 10.1097/MD.0000000000002414 PMC4718245 26765419

[84] Alfieri, F. M. et al. Effectiveness of an exercise program on postural control in frail older adults. *Clin. Interv Aging*. **7**, 593–598. 10.2147/CIA.S36027 (2012). 23269865 10.2147/CIA.S36027 PMC3529636

[85] Manor, B., Lipsitz, L. A., Wayne, P. M., Peng, C. K. & Li, L. Complexity-based measures inform Tai chi’s impact on standing postural control in older adults with peripheral neuropathy. *BMC Complement. Altern. Med.* **13**, 87. 10.1186/1472-6882-13-87 (2013). 23587193 10.1186/1472-6882-13-87 PMC3640896

[86] Kozlovskaya, I. B. et al. Russian countermeasure systems for adverse effects of microgravity on Long-Duration ISS flights. *Aerosp. Med. Hum. Perform.* **86**, A24–A31. 10.3357/AMHP.EC04.2015 (2015). 26630192 10.3357/AMHP.EC04.2015

[87] Fukagawa, N. K., Wolfson, L., Judge, J., Whipple, R. & King, M. Strength is a major factor in balance, gait, and the occurrence of falls. *Journals Gerontol. Ser. A: Biol. Sci. Med. Sci.* **50**, 64–67 (1995). 10.1093/gerona/50a.special_issue.64 7493221

[88] Abedi, H. et al. Non-contact, non-visual, multi-person hallway gait monitoring. *Sci. Rep.* **15**, 31166. 10.1038/s41598-025-97757-y (2025). 40851093 10.1038/s41598-025-97757-y PMC12375790

[89] Dupui, P., Montoya, R., Costes-Salon, M., Séverac, A. & Güell, A. Balance and gait analysis after 30 days-6 degrees bed rest: influence of lower-body negative-pressure sessions. *Aviat. Space Environ. Med.* **63**, 1004–1010 (1992). 1445150

[90] Clement, G. et al. Centrifugation as a countermeasure during bed rest and dry immersion: what has been learned? *J. Musculoskelet. Neuronal Interact.* **16**, 84–91 (2016). 27282452 PMC5114351

[91] Viseux, F. et al. How can the stimulation of plantar cutaneous receptors improve postural control? Review and clinical commentary. *Neurophysiol. Clin.* **49**, 263–268. 10.1016/j.neucli.2018.12.006 (2019). 30639034 10.1016/j.neucli.2018.12.006

[92] Shishkin, N., Kitov, V., Sayenko, D. & Tomilovskaya, E. Sensory organization of postural control after long term space flight. *Front. Neural Circuits*. **17**, 1135434. 10.3389/fncir.2023.1135434 (2023). 37139078 10.3389/fncir.2023.1135434 PMC10149828

[93] Ried-Larsen, M., Aarts, H. M. & Joyner, M. J. Effects of strict prolonged bed rest on cardiorespiratory fitness: systematic review and meta-analysis. *J. Appl. Physiol. (1985)*. **123**, 790–799. 10.1152/japplphysiol.00415.2017 (2017). 28705999 10.1152/japplphysiol.00415.2017

[94] Smith, M. G., Kelley, M. & Basner, M. A brief history of spaceflight from 1961 to 2020: an analysis of missions and astronaut demographics. *Acta Astronaut.* **175**, 290–299. 10.1016/j.actaastro.2020.06.004 (2020). 32801403 10.1016/j.actaastro.2020.06.004 PMC7422727

[95] Oksuz, E., Onat, E., Shahzadi, A., Yazici, Z. & Cetin, C. Evaluation of demographic characteristics, and general disease state of patients affliated with home health care unit of Malatya state hospital. *North. Clin. Istanb*. **1**, 166–172. 10.14744/nci.2014.14633 (2014). 28058324 10.14744/nci.2014.14633 PMC5175036

[96] Ikeda, T., Oka, S., Tokuhiro, J., Suzuki, A. & Matsuda, K. Short-Term cast immobilization of a unilateral lower extremity and physical inactivity induce postural instability during standing in healthy young men. *Healthc. (Basel)*. 11. 10.3390/healthcare11182525 (2023). 10.3390/healthcare11182525 PMC10531174 37761723

[97] Vieira, E. R., Palmer, R. C. & Chaves, P. H. Prevention of falls in older people living in the community. *BMJ* **353**, i1419 (2016). 10.1136/bmj.i1419 10.1136/bmj.i1419 27125497

[98] Nevitt, M. C., Cummings, S. R., Kidd, S. & Black, D. Risk factors for recurrent nonsyncopal falls. A prospective study. *JAMA* **261**, 2663–2668 (1989). 2709546

[99] Schmid, M., Casabianca, L., Bottaro, A. & Schieppati, M. Graded changes in balancing behavior as a function of visual acuity. *Neuroscience* **153**, 1079–1091. 10.1016/j.neuroscience.2008.03.024 (2008). 18440719 10.1016/j.neuroscience.2008.03.024

[100] Talbot, L. A., Musiol, R. J., Witham, E. K. & Metter, E. J. Falls in young, middle-aged and older community dwelling adults: perceived cause, environmental factors and injury. *BMC Public. Health*. **5**, 86. 10.1186/1471-2458-5-86 (2005). 16109159 10.1186/1471-2458-5-86 PMC1208908

